# Vowel production of Mandarin-speaking hearing aid users with different types of hearing loss

**DOI:** 10.1371/journal.pone.0178588

**Published:** 2017-06-02

**Authors:** Yu-Chen Hung, Ya-Jung Lee, Li-Chiun Tsai

**Affiliations:** Speech and Hearing Science Research Institute, Children’s Hearing Foundation, Taipei City, Taiwan; Northwestern University, UNITED STATES

## Abstract

In contrast with previous research focusing on cochlear implants, this study examined the speech performance of hearing aid users with conductive (n = 11), mixed (n = 10), and sensorineural hearing loss (n = 7) and compared it with the speech of hearing control. Speech intelligibility was evaluated by computing the vowel space area defined by the Mandarin Chinese corner vowels /a, u, i/. The acoustic differences between the vowels were assessed using the Euclidean distance. The results revealed that both the conductive and mixed hearing loss groups exhibited a reduced vowel working space, but no significant difference was found between the sensorineural hearing loss and normal hearing groups. An analysis using the Euclidean distance further showed that the compression of vowel space area in conductive hearing loss can be attributed to the substantial lowering of the second formant of /i/. The differences in vowel production between groups are discussed in terms of the occlusion effect and the signal transmission media of various hearing devices.

## Introduction

Hearing loss adversely affects speech perception, leading to specific speech characteristics. Successful communication through spoken language requires mutual understanding of verbal signals. Disabilities in auditory function in people with hearing loss often result in atypical and ultimately less intelligible speech, which lead to substantial difficulties in communicating effectively through spoken words [1]. Especially, as communication is essential to social interactions and healthy relationships, a recent study has pointed out that intelligible speech is a relevant ability for children with hearing loss to maintain their social status and enrollment in hearing and speaking environments [2]. Therefore, understanding the strengths and weaknesses of their speech performance is crucial for developing intervention strategies to improve the intelligibility. Numerous studies have shown that people with hearing loss often nasalise speech sounds [3–5], and exhibit speech flow disruptions, resulting in abnormal speech rhythm. Moreover, their speaking rate is generally reduced as a result of prolonged production of speech segments and slow articulatory transitions [6–10].

Regarding segmental units, people with hearing loss frequently struggle to distinguish sounds with similar phonetic features, such as voiced–voiceless cognate pairs, which have identical places and manners of articulation and only differ from each other in vocal vibration, or fricative and affricate cognates, as the latter begin as plosives and release into fricatives. Consequent common errors include deletions of initial and final consonants [8,11–13], simplifications of consonant clusters [14,15], and substitutions of one consonant for another [12,16–19]. In contrast to consonants that often share articulatory features and are shaped by minimal active movements, vowels are normally formed by positioning the tongue and lips in various ways [20]. As a consequence, vowels are usually produced more accurately because of their unique articulatory position and the acoustic intensity involved in their production [21,22]. Nevertheless, certain errors have still been frequently observed in people with hearing loss, such as vowel substitution [3,15,23], neutralisation [11,15,17], and diphthong misarticulation [3,6,11,15,17].

Additional studies have been conducted to quantitatively investigate the speech intelligibility of people with hearing loss. Speech intelligibility indicates the degree to which a message delivered by a speaker is comprehensible [24]. It can be conventionally measured either by calculating the accuracy of words or phonemes in a written task, wherein the listener writes down what they understood from a speech sample [25], or by using a rating scale to judge speech, wherein the listener estimates the proportion of the presented speech that they understood [26,27]. Because intelligible speech is often considered the ultimate goal for children with hearing loss [25], numerous studies have been conducted to study the possible factors contributing to comprehensible speech. For example, hearing capacity and the length of hearing aid (HA) use have been found to positively correlate with speech intelligibility [15,28–30]. Moreover, Markides [31] observed that children fitted with HAs before the age of 6 months produced more comprehensible speech than children fitted at later ages.

Similarly, with the advent of cochlear implants (CIs), many researchers have shifted their attention towards the questions of whether and how the signals conveyed by electrical stimulation might affect the quality of speech perception and production in CI recipients. These studies have generally concluded that early implantation yields more intelligible verbal expression than later implantation does [32–36]. Speech intelligibility has been found to improve gradually over time, especially when users are implanted with CIs at younger ages [36–41].

In addition to the transcription or rating of speech materials, vowel space area (VSA) also functions as a useful index for assessing speech performance. Articulatory working space is a graphic display of the first (F1) and second (F2) formants. The value of F1 varies with the height of the tongue, whereas the value of F2 is mostly determined by tongue retraction (back or front position). Crucially, the VSA has been shown to positively correlate with speech intelligibility scores, not only in people with typical development [42–46] but also in specific populations such as people with speech disorders [47–50] and people with hearing loss [51,52]. A larger VSA is indicative of clearer speech. The performance of CI speakers has been the focus of numerous studies on the acoustic analysis of VSA in people with hearing loss. The majority of studies have shown that CI children exhibit a smaller VSA than children with normal hearing [53–58]. Others have reported the opposite, namely that the vowel space performance of CI children approximates that of their peers with normal hearing [59]. However, this discrepancy might be partially attributable to demographic differences. For example, in comparison to other studies [53,54,56,57], most CI speakers in the study by Uchanski and Geers [59] had received implantation at an earlier age (i.e., <3 years) and their duration of CI use was longer as well (i.e., 4–6 years), which is likely what led to them having a similar vowel performance to that of the control group [32–36].

In addition to calculating the area of vowel space, the measure of formant values facilitates the assessment of the acoustic and articulatory features of each individual speech element. More crucially, by combining the computation of the VSA and the evaluation of formant patterns, researchers are able to identify the possible origins of discrepancies in speech performance. For example, the F2s of corner vowels in CI speakers have been found to be more divergent and generally lower than those in speakers with normal hearing, resulting in horizontally compressed vowel space [29,54,55,57,60].

Although a large body of work exists on the speech performance of CI speakers, only a few studies have focused on direct acoustic comparisons between CI and HA users. Horga and Liker [53] found that CI speakers achieved higher vowel quality than their HA counterparts, particularly in front and back vowels. However, opposite findings were presented by Verhoeven et al. [60]; in relation to the HA speakers, the CI speakers displayed greater overlaps between vowel categories, yielding reduced vowel contrasts and speech intelligibility. However, this disagreement between results could be a reflection of the severity of hearing loss in the HA samples. The HA participants in Horga and Liker [51] were all profoundly hearing-impaired, whereas those in Verhoeven et al. [58] had only mild-to-moderate hearing loss. The latter group with milder hearing loss might show higher overall speech outcomes, since children with more residual hearing have generally been reported to have higher performance in speech perception and production [61–63].

The aforementioned studies have shed light on differences in acoustic–articulatory performance between two groups using various hearing devices with distinct sound transmission principles; however, to the best of our knowledge, little research has targeted HA users alone. There is a large number of HA users worldwide [64], whose types of hearing loss can be categorised into three subgroups according to the damaged part of the auditory system: conductive hearing loss, sensorineural hearing loss, and mixed hearing loss. Conductive hearing loss is frequently caused by damage or an obstruction in the outer or middle ear, leading to problems in conducting airborne sounds. Thus, bone-conducting or bone-anchored HAs are used to transfer sound waves into sound vibrations, which further move across the skull into the inner ear. By contrast, the main cause of sensorineural hearing loss lies in the inner ear or along the auditory nerve, and patients normally wear air-conducting HAs. Mixed hearing loss refers to cases when conductive hearing loss occurs in conjunction with sensorineural hearing loss.

The quality of sound differs when it is transferred through different media [65]. For instance, signals delivered by HAs sound different to those transmitted by CIs. This is because CIs transform sounds into electrical current that directly stimulates the auditory nerve [66,67]. Similarly, because different hearing loss types are usually associated with different transmission paths, people with conductive hearing loss who use bone-conducting HAs are likely to perceive signals differently to people with sensorineural hearing loss who wear air-conducting HAs.

To shed greater light on the extent to which hearing loss type affects speech performance, the present study included all three types of hearing loss and examined their articulatory working space and acoustic-articulatory quality by using Mandarin Chinese corner vowels. Corner vowels are frequently used to calculate the size of the articulatory working space, because they represent the most extreme positions of the tongue, defining the boundary of the area within which vowels can be produced [50]. Because perceptual variation affects the quality of speech production [68–70], we expected to observe divergent speech quality across different types of hearing loss. In particular, we expected to observe media-dependent and therefore frequency-dependent effects on articulatory working space. Because bone more effectively transmits low-frequency sound than air does [65], people with conductive hearing loss wearing bone-conducting HAs might perceive sounds differently than people with the other two types of hearing loss. For example, the low-frequency components in the sound /i/ might overpower high-frequency components when the sound is transmitted through bone, in what is known as the occlusion effect. This results in a less typical acoustic representation of /i/, which in turn presumably affects speech production. Examining articulatory performance would hence provide us with insights into the perceived quality of sound associated with different signal transmission paths (i.e., airborne or bone-borne). Furthermore, the findings have valuable clinical implications regarding concerns such as HA fitting strategies and guidelines for speech training. Finally, regardless of the type of hearing loss, articulatory distortions should mostly occur in the back vowels (e.g., /i/) because of the lack of visible articulatory movements [54,55,57].

## Materials and methods

### Participants

Twenty-eight speakers with mild to moderately severe bilateral hearing loss participated in this study. They were all prelingually deaf and had been enrolled in auditory–verbal therapy programmes for an average of 4 years. At the time of the experiments, they mainly used oral communication. According to the type of hearing loss, they were assigned to three subgroups: conductive hearing loss (COND), mixed hearing loss (MIX), and sensorineural hearing loss (SNHL).

The COND group contained 11 participants (mean age: 9 years) whose hearing conditions resulted from congenital aural atresia or microtia, and who wore either bone-conducting or bone-anchored HAs. The MIX and SNHL groups consisted of 10 (mean age: 13 years) and seven (mean age: 14 years) participants, respectively. They were all fitted with air-conducting HAs. More auditory-related details of the participants in the hearing loss groups (HL) are summarised in Table 1. In addition, 26 speakers (mean age: 28 years) with normal hearing were recruited in this study as the control group (NH). All the participants were monolingually raised native speakers of Mandarin Chinese in Taiwan. None of the participants had cleft lip or cleft palate.

**Table 1:** Speaker demographics.

Type of hearing loss	Subj.	Gender	Chronological age (yr)	Hearing age (yr)	Intervention duration (yr)	PTA
Conductive	C01	F	14.8	13.5	0.8	51
	C02	F	7	6.6	5.9	63
	C03	F	7.9	7.5	7.8	57
	C04	F	8.8	7.5	6.4	82
	C05	M	10.5	9.7	2.5	28
	C06	M	11.4	11.1	3.3	50
	C07	M	11.6	8.5	3.1	48
	C08	M	8.1	7.7	6.8	58
	C09	M	7	5.7	5.8	57
	C10	M	7	6.3	5.4	76
	C11	M	9	8.1	7.3	32
Mixed	M01	F	11.5	7.5	2.8	55
	M02	F	10.8	10.3	5.8	31
	M03	M	15.5	9.2	0.7	48
	M04	M	16	11.6	1	69
	M05	M	10.2	9.5	3.4	41
	M06	M	10.3	4.8	4.6	55
	M07	M	10.8	7.4	5.3	55
	M08	M	21.2	17	3.4	54
	M09	M	11.8	7.8	4.4	48
	M10	M	10.6	5.9	4.5	59
Sensorineural	S01	F	10.3	7.2	3.6	40
	S02	F	13.3	9	3.4	45
	S03	F	19.5	13.3	0.8	70
	S04	F	10	6.9	5.4	53
	S05	M	11.9	7.5	0.4	66
	S06	M	19.1	14.1	1.1	75
	S07	M	15.7	12.2	5.1	49

Note. Subj. = subject number; M = male; F = female; PTA = the pure-tone average. Intervention = Auditory-verbal therapy.

Although age has been found to affect the size of vowel space [71], we intentionally included adult NH speakers as the control group because they have more skilled articulator movements and their speech production is more likely to meet the characteristics of clear speech per definition [72,73]. Therefore, having an adult control group allows us to highlight the differences in speech intelligibility between the HL groups and the typical speech sample.

### Ethics statements

Information about the experiment was provided and written informed consent was collected prior to participation. Parents or guardians were asked to provide written informed consent on behalf of participants who were minors. All participants were compensated financially. This study was conducted according to the principles expressed in the Declaration of Helsinki and the procedure was approved by the Institutional Review Board of the Chang Chung Medical Foundation in Taiwan.

### Speech materials and recording procedures

The speech material comprised the phonetic chart for Mandarin Chinese, including three corner vowels /a, i, u/, and 34 other phonetic elements as fillers, ensuring that the participants remained naïve with respect to the purpose of the recording.

Prior to the recording session, participants in the HL groups were asked to complete a pure-tone audiometric test carried out by a licensed audiologist in a sound-treated room. Following the audiometric test, each speaker was provided with speech material written in Mandarin Phonetic Symbols, also known as Zhuyin fuhao, which are officially used in Taiwan for the phonetic transcription of Chinese sounds. During recording, the distance between the microphone and the speaker was maintained at approximately 15 cm. The speakers were instructed to produce each speech sound three times in isolation in a neutral voice at a normal speech rate and not to purposely exaggerate their articulation. The speech materials were recorded using Praat (Version 6.0.19) [74], and directly stored in a laptop (HP Probook 4421s) at a sampling rate of 44100 Hz with a resolution of 16 bits.

### Acoustic analysis

**Fig 1:**
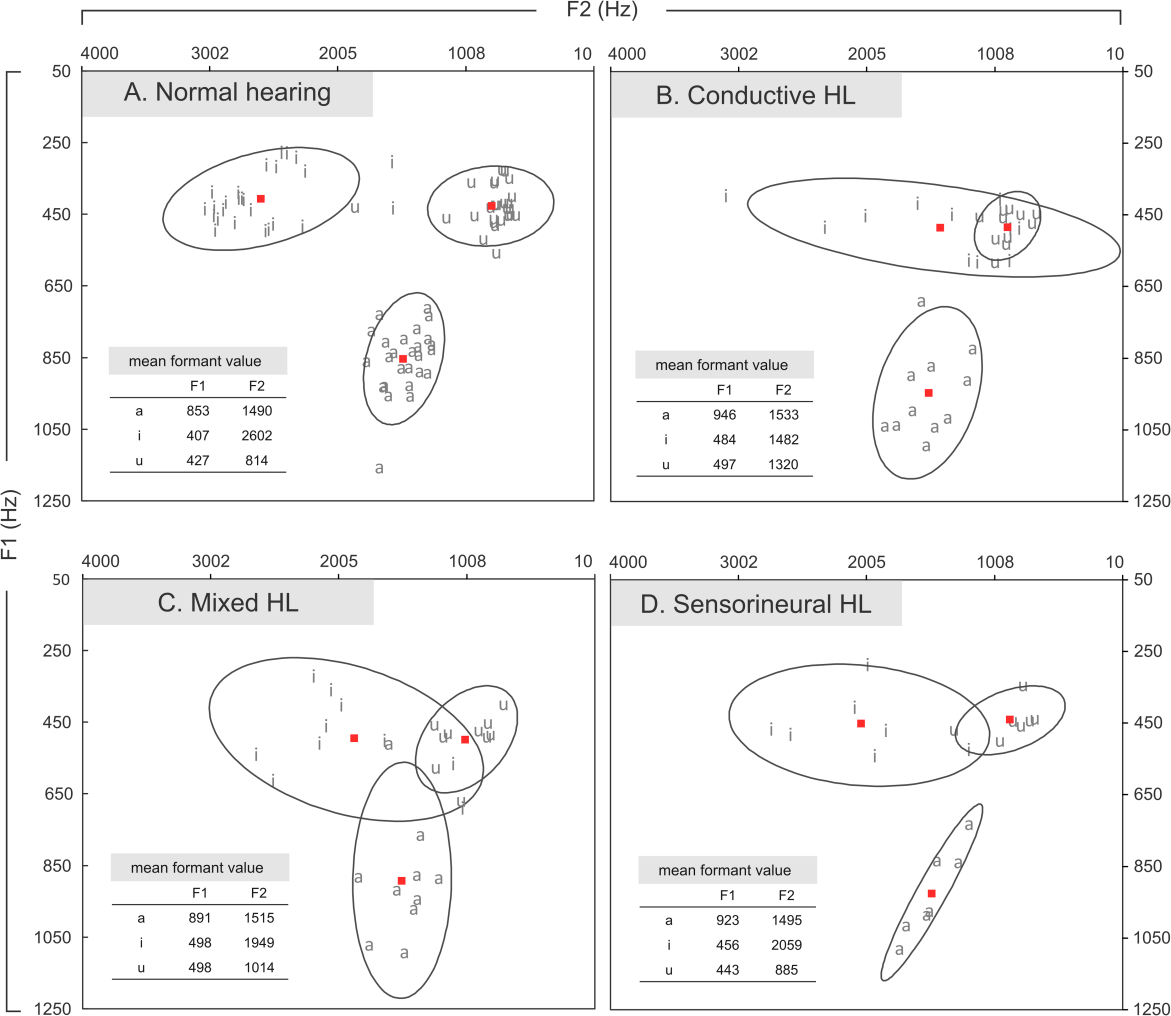
Average values of the first (F1) and second (F2) formant for each group. The ellipses were drawn with two standard deviations from the mean of each vowel, incorporating approximately 95% of the data points. Each symbol (a, u, i) represents the average F1 and F2 value for each speaker, and the red squares represent the central coordinate (i.e., the mean F1 and F2 values) for each ellipse.

F1 and F2 were measured at the steady-state segment of each corner vowel using Praat. The average values for F1 and F2 in Hz were first calculated for each speaker and each vowel, which were then used to obtain the grand average formant frequencies for each subgroup. According to the results of the acoustic measurements, each vowel was plotted on a chart with F1 on the y-axis and F2 on the X-axis to reflect its position in the oral cavity. Fig 1 illustrates the scatter plots of F1 and F2 for each group. The vowel ellipse has been used to indicate articulatory variability [60,75,76]. The elliptical range was drawn with two standard deviations (SDs) from the mean of each vowel, averaged over all participants in each group. The semimajor and semiminor of the ellipse represented either the SDs of the mean F1 or the mean F2. To determine the interspeaker variability for each vowel, the area of ellipse was calculated, with the centre coordinates corresponding to the average values of F1 and F2 as a reference [57,77]. Larger areas were indicative of enhanced interspeaker variability [60,75,76].

### Vowel space area

To obtain the VSA produced by each group, the following formula (1) was applied [50]:

$$\frac{\left[\text{F}1\text{i}\times \left|\text{F}2\text{a}-\text{F}2\text{u}\right|+\text{F}1\text{a}\times \left|\text{F}2\text{u}-\text{F}2\text{i}\right|+\text{F}1\text{u}\times \left|\text{F}2\text{i}-\text{F}2\text{a}\right|+\text{F}1\text{u}\times \left|\text{F}2\text{i}-\text{F}2\text{a}\right|\right]}{2}$$ (1)

F1i represents the F1 value of the vowel /i/ and F2a represents the F2 value of the vowel /a/. The same principle applies to other symbols in the formula. A larger VSA indicates more intelligible speech [42,46,78].

### Euclidean distance

The Euclidean distance in the F1–F2 plane was then measured to quantitatively compare the acoustic differences between the HL subgroups and the NH group across three corner vowels. The distance is calculated using formula (2), where F1_HLsp_ and F2_HLsp_ are the mean F1 and F2 value of a given vowel produced by a certain HL speaker (e.g., the average of the three tokens of /a/ produced by a speaker with conductive hearing loss), and $F1{\bar{M}}_{\text{NH}}$ and $F2{\bar{M}}_{\text{NH}}$ are the grand mean F1 and F2 values of the same vowel of the NH group [55,57,58].

$$\sqrt{{\left(\text{F}1_{\text{HSLP}}-\text{F}1{\bar{M}}_{\text{NH}}\right)}^{2}+{\left(\text{F}2_{\text{HSLP}}-\text{F}2{\bar{M}}_{\text{NH}}\right)}^{2}}$$ (2)

A greater Euclidean distance indicates more dissimilar vowel quality between participants in the HL and NH groups [57].

## Results

### Demographic factors

We first evaluated the demographic variables that could affect the quality of vowel production among HL subgroups. Because the range of each variable was rather large, as shown in Table 1, Kruskal–Wallis tests were performed to detect whether the median of each variable differed between groups. No significant differences were found between subgroups in the pure-tone average threshold in the better ear (*H*(2) = 3.701, *p* = .854) or in relation to hearing age (i.e., duration of HA use) (*H*(2) = 5.742, *p* = .670) or duration of intervention (*H*(2) = 7.563, *p* = .114). The only significant difference was that for chronological age (*H*(2) = 11.201, *p* = .021). A follow-up test using the Bonferroni approach was conducted to evaluate pairwise differences among the three groups. A significant difference was observed between the COND and SNHL groups (*H*(2) = 29.267, *p* = .011), indicating that the COND group was younger than the SNHL group. No differences were observed between the other groups (MIX vs SNHL: *H*(2) = 2.629, *p* = 1.0; COND vs. MIX: *H*(2) = −7.527, *p* = .109)

### Speech intelligibility: Vowel space area

**Fig 2:**
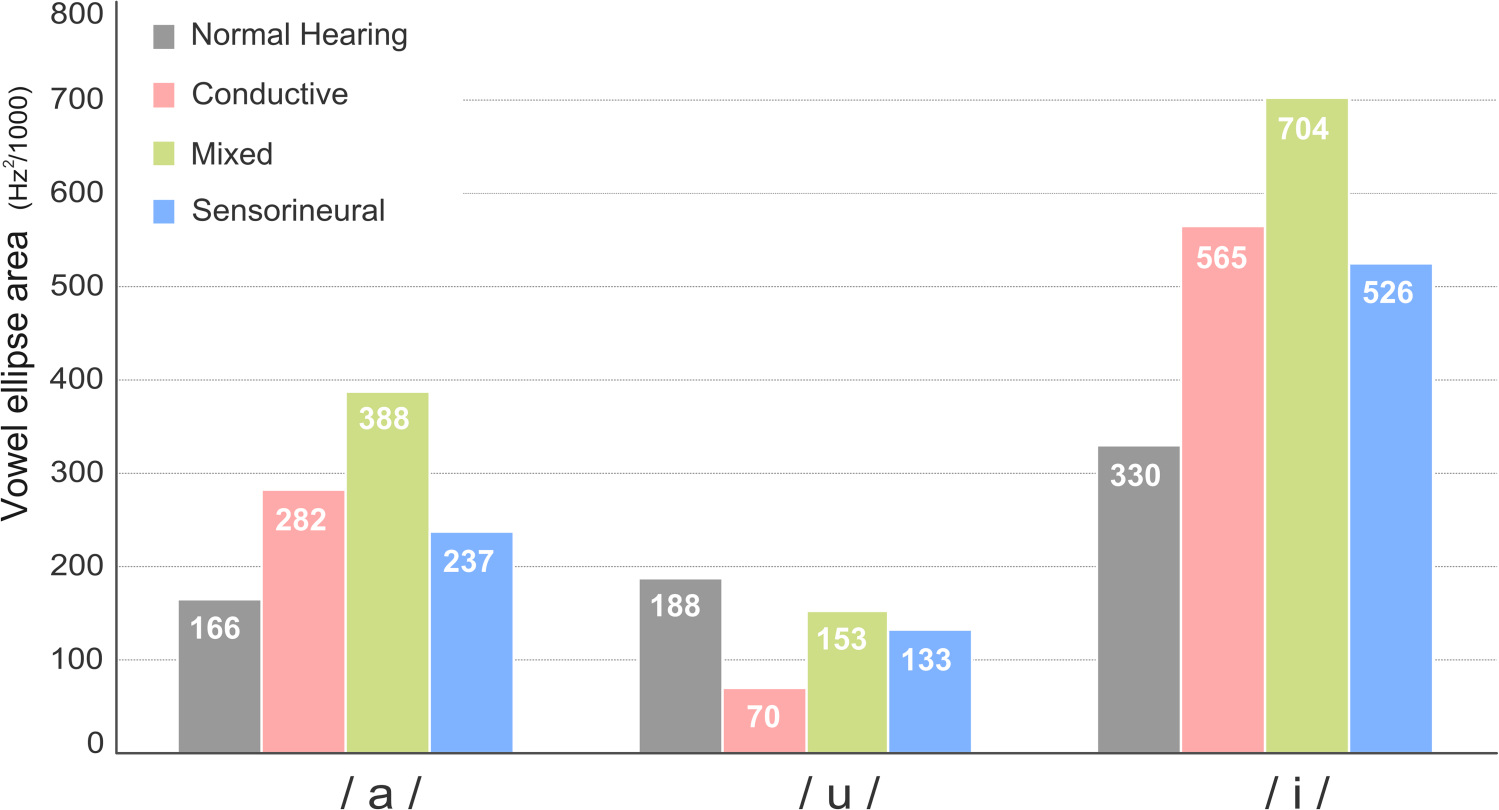
Vowel ellipse area for each Mandarin Chinese corner vowel in each group.

On visual inspection of Fig 1, /i/ appeared to be the least stable sound across all vowels and groups, reflected in its greater ellipses. In particular, the F2s of /i/ in the COND group were generally shorter than those of other groups, resulting in a substantial degree of overlap in the formant frequency patterns between /u/ and /i/. A direct comparison between the values of vowel ellipse areas across groups appears to support the results of the visual inspection, as displayed in Fig 2. Namely, regardless of the type of hearing loss, the sound /i/ generally exhibited the highest interspeaker variability among three corner vowels, reflected in the largest ellipse area.

**Fig 3:**
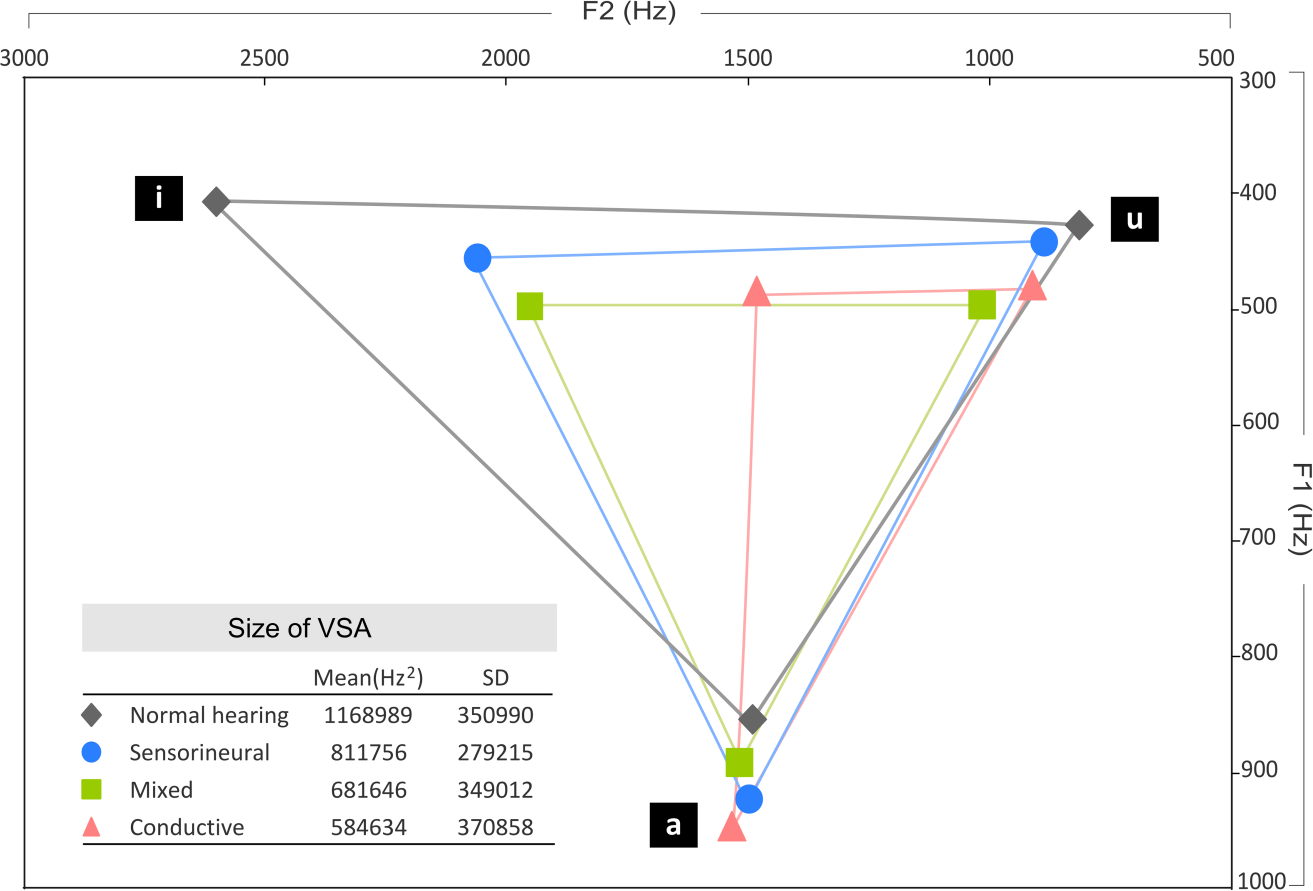
Mandarin Chinese vowel formant space for each group.

Fig 3 presents the VSA value for each group. First, a t-test was conducted to compare the VSA difference between the NH and HL groups. The results revealed that the NH speakers produced significantly larger VSAs than the HL speakers did (*t*(52) = 5.227, *p* < .0001, Cohen’s *d* = 1.50). This correlates with the results of previous studies [53–58], implying that people with hearing loss generally exhibit reduced speech intelligibility in comparison with people with normal hearing.

A one-way ANOVA test was then carried out to further examine whether any difference existed in VSA performance between each HL subgroup and the NH group. With a main effect of groups (*F*(3,50) = 9.685, *p* < .0001; *η*^2^ = 0.91), post hoc comparisons using Dunnett’s T3 procedure showed that although no VSA difference was apparent between the SNHL and NH groups (*F*(1, 31) = 6.172, *p* = .080), both the COND and MIX groups exhibited significantly more compressed VSAs than the NH group did (NH vs COND: *F*(1, 35) = 20.736, *p* = .002; NH vs MIX: *F*(1, 34) = 13.965, *p* = .010). Moreover, no significant VSA difference was found between SNHL and COND (*F*(1, 16) = .379, *p* = .611), SNHL and MIX (*F*(1, 15) = .662, *p* = .094), or COND and MIX (*F*(1, 19) = .837, *p* = .988).

### Vowel quality: Euclidean distance

**Fig 4:**
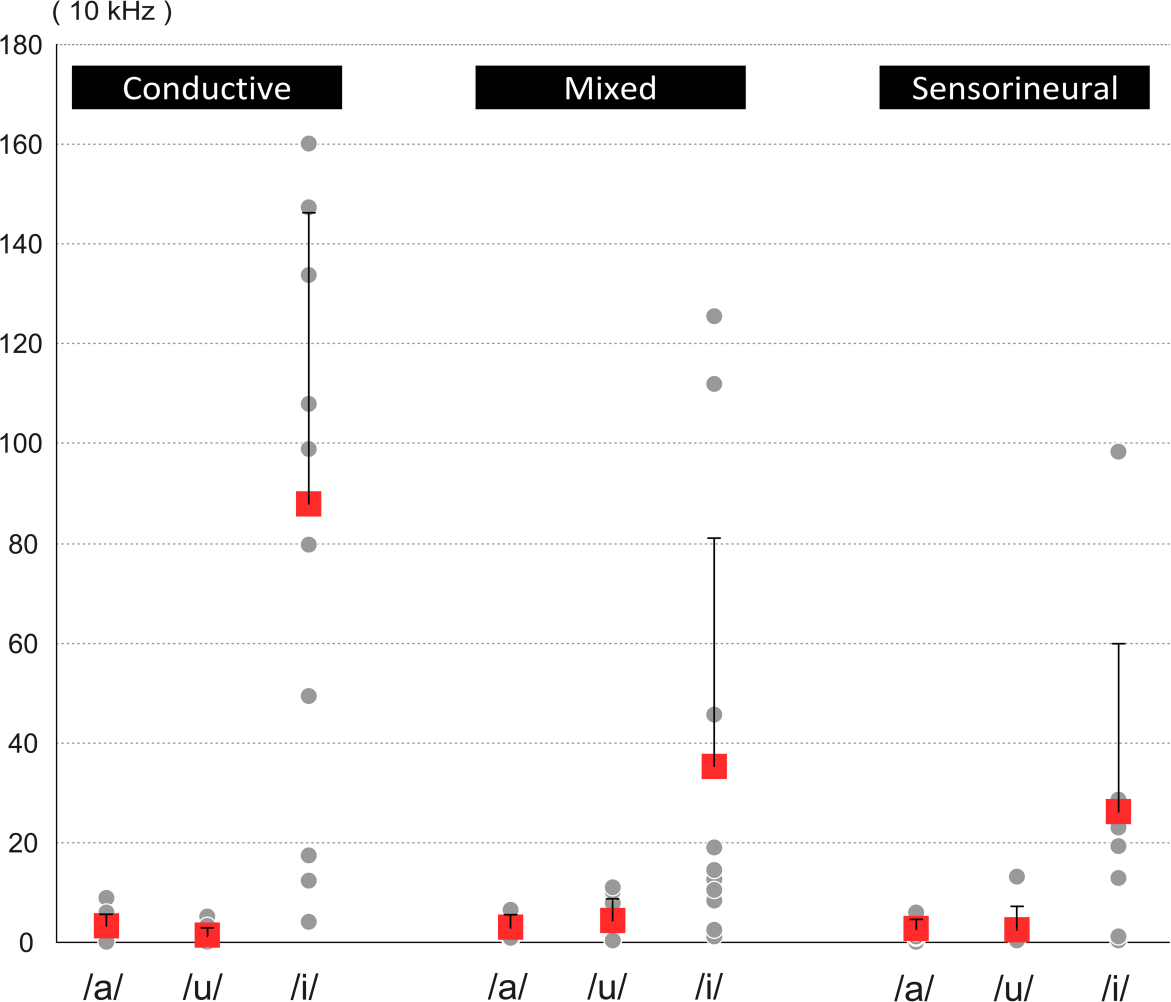
Average Euclidean distance of each Mandarin corner vowel between each hearing loss subgroup and the normal hearing group. Each point represents one speaker. Red squares stand for the average distance value and error bars represent standard deviations.

To examine the mean Euclidean distance between each HL subgroup and the NH group across the corner vowels, repeated measures ANOVA was performed for the COND, MIX, and SNHL groups separately. Greater Euclidean distance indicated more acoustic dissimilarity between the target vowel in a given HL subgroup and the NH group. As shown in Fig 4, a significant main effect was found for the COND group (*F*(2,20) = 22.64, *p* < .0001, *η*^2^ = 0.69), and the post hoc t-tests showed that /i/ had a significantly greater distance than /a/ and /u/ did (*p*s = .001), indicating that speakers with COND had difficulty pronouncing /i/ correctly. By contrast, no significant effect was found in the MIX and SNHL groups, suggesting that their vowel qualities were relatively similar to those of the NH group.

To examine which formant feature was mainly responsible for the articulatory inconsistency of /i/ in the COND group, we calculated the differences between the COND and NH groups at the F1 and F2 formants, respectively. The statistical analysis confirmed the visual inspection of Fig 3; both the F1 and F2 values in the COND were significantly different from those in the NH group. Notably, in contrast with the relatively minor F1 differences (*t*(35) = 3.034, *p* = .005, Cohen’s *d* = 1.03), a substantial F2 discrepancy was found (*t*(35) = 6.174, *p <* .0001, Cohen’s *d* = 2.09), indicating that people with conductive hearing loss tended to retract the tongue farther backward while producing the sound /i/.

Overall, speakers with COND or MIX exhibited significantly smaller VSAs relative to the NH group, indicating reduced speech intelligibility. Specifically, in contrast to the relatively homogeneously centralised VSA in the MIX group, the more compressed articulatory working space in the COND group seemed to result from a less accurate articulation of /i/ with a substantial backward displacement of the tongue body, as mirrored in the significantly lower F2 value [20].

## Discussion

The main aim of the present research was to explore whether speakers with different types of hearing loss produce vowels differently than speakers with normal hearing. Similarly to other investigations [53–58], the present results showed a generally more compressed articulatory working space in the HL groups than in the NH group (Fig 3), indicating less intelligible speech. However, a fine-grained analysis comparing vowel production between different HL subgroups revealed that people with SNHL performed similarly to the NH group, whereas the COND and MIX groups showed significantly reduced VSA. Unlike previous studies showing that hearing aid users generally have a smaller VSA than people with normal hearing [53,60], the present results suggest otherwise. Namely, the hearing aid users with SNHL exhibited VSA similar to that of their hearing counterparts. Although Verhoeven et al. [51] and Horga and Liker [58] have also collected speech data from conventional hearing aids users (i.e. air-conduction), they have not reported whether their samples included both SNHL and MIX groups or only an SNHL group. As the present results demonstrated, vowel quality tended to differ between these two types of hearing loss. The contradictory findings might therefore be attributed to the effect of hearing loss types. Crucially, this study showed that people with SNHL might eventually achieve a comparable level of speech intelligibility to people with typical hearing. However, other factors that might contribute to intelligible speech such as hearing age or the duration of intervention must be examined more thoroughly in future research.

Turning to the findings of significantly more reduced VSA in the COND and MIX groups, the calculation of the Euclidean distance further demonstrated that, in contrast to a centralised VSA in the MIX group, the heterogeneously shrunken VSA in the COND group was mainly caused by a significant divergence in /i/ production (Fig 4). More specifically, the analysis of acoustic profiles suggested that the reduction of F2 in /i/ was greater than F1, causing a horizontal shift of VSA towards the back of the cavity.

Previous research has claimed that the unstable production of /i/ along the front-back dimension is induced by less visible biofeedback involving tongue movements [54,55,57]. Compared to other vowels with prominent visual cues, such as openness (e.g., /a/) and roundedness (e.g., /u/), the lack of visibility impedes children with hearing loss in accurately acquiring the place of articulation. However, this assumption of articulatory visibility would only apply to an observation of overall less consistent performance of /i/ in all HL subgroups (Fig 3). By contrast, our data shows a highly specific effect, with F2 lowering in /i/ only for the COND group. Thus, this effect must be attributed to other causes.

The shrunken VSA induced by significantly centralised /i/ could simply reflect age difference, because the average chronological age of the COND group was younger than that of other subgroups. However, VSA has been shown to negatively correlate with chronological age; namely, older speakers display smaller VSA [71,79,80]. Therefore, the VSA reduction found in the COND group was not a result of factors related to chronological age.

By contrast, we suggest that the present result was more likely to be a consequence of signal conduction methods. Bone is more capable of conducting lower frequencies than air is; sounds transmitted through bone are therefore perceived to have deeper and lower tones [65]. All speakers with conductive hearing loss in the present study had either congenital aural atresia or microtia, and all were fitted with bone-conducting HAs. The closing at the outer ear pathway could cause the occlusion effect, which increases sound at 500 Hz up to 25 dB or at 200 Hz up to 40 dB [81]. In contrast to /a/ and /u/ with both formants located in the low-to-mid frequency range, the vowel /i/ is more dispersed in the phonetic space, with F1 in low frequencies and F2 in high frequencies [77]. In other words, when wearing bone-conducting HAs, the high-frequency component of the transmitted signal is likely to be masked, at least to some extent, by the low-frequency sounds, leading to perceptual distortion. Crucially, it has been assumed that perceptual experiences can help shape and establish internal acoustic representations of target speech sounds, which are stored in the memory, serving as a guide for speech production. For example, by observing developmental changes in vowel production, a past study found that infants were able to access phonetic category information and vocally imitate the sounds they heard [82]. Interestingly, a similar effect was also reported in second language learning, namely that the reinforcement of perceptual training could significantly improve performance in speech production, providing further support for the link between speech perception and production [83]. As a result, a constant perception of distorted sound would presumably affect the sensorimotor learning of speech, leading to poor production. Thus, the occlusion effect induced by bone-conducted stimulation probably caused a more substantial reduction of F2 in /i/ for participants with conductive hearing loss than for those with mixed or sensorineural hearing loss, because the /i/ sound they perceived would be more dominant at lower frequencies. However, future research is required to clarify whether the occlusion effect has an impact on overall speech performance in this population.

Although this study sheds light on speech intelligibility and vowel quality between people with different types of hearing loss, it has limitations that must be addressed. First, our findings are derived from a relatively small sample size. Therefore, caution is advisable when generalizing the research findings to a larger population of interest. Second, the participants in the current study were instructed to read aloud isolated phonemes, which might be considered less natural and rather monotonous in comparison to spontaneous speech. In particular, it has been found cross-linguistically that the coarticulatory effects involved in continuous speech may affect vowel production, probably leading to different intelligibility outcomes than phonemes pronounced in isolation [84–86]. In order to fully understand differences in natural speech performance between people with different types of hearing loss, future research should focus on intelligibility assessment for spontaneous speech.

Nevertheless, the present study was, to our knowledge, the first to report on how different types of hearing loss with distinct transmission paths affect speech performance. The findings suggested that bone-conducting HAs, typically used by people with conductive hearing loss, seemed to transmit signals differently in a frequency-dependent manner, resulting in low frequencies overpowering high frequencies. This in turn may affect speech production, with reduced intelligibility in the high-frequency range. Clinically, the current results highlight the role of the occlusion effect. When assessing the auditory performance of patients wearing bone-conducting HAs, the occlusion effect should be carefully considered to avoid signal distortions that may degrade sound quality. Speech therapists should also consider the possibility that reduced speech intelligibility might be the result of excessive low-frequency amplification.

## Supporting information

S1 File Minimal dataset table. The PTA and the formant values for each participant in each group.
(XLS)
